# Hepatitis A Virus Infection in Cynomolgus Monkeys Confounds the Safety Evaluation of a Drug Candidate

**DOI:** 10.1177/10915818241237992

**Published:** 2024-03-19

**Authors:** Chris J. Powell, John C. Kapeghian, John C. Bernal, John R. Foster

**Affiliations:** 1 MRC Toxicology Unit, 2152 University of Cambridge, Cambridge, UK; 2 Preclinical Safety Associates, LLC, The Woodlands, TX, USA; 3 CORES Veterinary Consulting, Walnut Grove, TX, USA; 4 ToxPath Sciences Ltd, Congleton, UK

**Keywords:** toxicology, hepatitis A virus, cynomolgus monkey, non-human primate, hepatotoxicity, hepatic pathology, safety evaluation, drug development

## Abstract

In a 3-month toxicity study in cynomolgus monkeys at a European contract laboratory, animals were infected with HAV, initially resulting in hepatic injury being incorrectly attributed to the test compound. Elevated serum ALT/AST/GLDH (5- to 10-fold) were noted in individual animals from all groups including controls, with no apparent dose, exposure, or time-related relationship. Liver histopathology revealed minimal to slight inflammatory cell accumulation in periportal zones of most animals, and minimal to slight hepatocyte degeneration/necrosis in 10/42 animals from all groups. As these findings were more pronounced in 6 drug-treated animals, including 2/6 in the low dose group, the draft report concluded: “*treatment-related hepatotoxicity at all dose levels precluded determination of a NOAEL*.” However, the unusual pattern of hepatotoxicity suggested a factor other than drug exposure might have caused the hepatic effects. Therefore, snap-frozen liver samples were tested for hepatitis viruses using a PCR method. Tests for hepatitis B, C, and E virus were negative; however, 20/42 samples were positive for hepatitis A virus (HAV). Infection was strongly associated with increased serum ALT/GLDH, and/or hepatocyte degeneration/necrosis. Re-evaluation of the study in light of these data concluded that the hepatic injury was not drug-related. A subsequent 6-month toxicology study in HAV-vaccinated cynomolgus monkeys confirmed the absence of hepatotoxicity. Identification of HAV infection supported progression of the drug candidate into later clinical trials. Although rarely investigated, subclinical HAV infection has occasionally been reported in laboratory primates, including those used for toxicology studies and it may be more prevalent than the literature indicates.

## Introduction and Background

Toxicology studies are normally conducted using healthy young animals. However, very occasionally, an outbreak of infectious disease has compromised experimental safety studies, for example, *Helicobacter spp* in studies sponsored by the US National Toxicology Program, Tyzzers disease (*Clostridium piliforme*) or Sialodacryoadenitis virus (a coronavirus) in rodents, and measles,^1-4^ or hepatitis viruses in primates.^5-8^ Hepatitis A virus (HAV) is a widely distributed, single stranded RNA picornavirus which infects humans and several primate species.^9^ It is highly contagious, transmitted via the oral faecal route, although usually causing only a mild, self-limiting, or subclinical infection.^6^ Hepatitis B virus (HBV) infection has also been reported in cynomolgus monkeys from Mauritius, with viral DNA detected in over 40% of liver samples in one study.^10^

While subclinical infections in toxicology studies are more difficult to detect unless animals are serologically screened, evidence that they have influenced experimental results sometimes emerges: for instance when immune function is evaluated in studies assessing the effect of protein or nucleic acid based therapies, or when the effects of therapeutic immune-response modifiers have been investigated.^11,12^ It is rare for the outcome of an experimental safety study to be confounded by infection, although it can have serious consequences when it does occur.^13^ Reflecting the need to control potentially confounding variables such as infection, most experimental animals used in biomedical studies are purpose-bred, reared under carefully controlled environmental conditions, and their health status thoroughly evaluated before they are considered suitable for use. Sentinel animal screening is a routine part of quality control processes and, in many cases, dogs and primates are vaccinated against high-risk pathogens.

The translational relevance of toxicology studies for humans is based on the premise that the experimental models are a reasonable proxy for the biological effects that would occur in humans, and within a study, the identification of treatment-related effects is normally underpinned by an exposure– (or dose) response relationship. Determining whether effects observed were the direct result of exposure to the test compound, and whether they are reliably human relevant, sometimes requires expert knowledge of the strengths and limitations of the experimental model and of the test methods employed. Confounding factors may include pre-existing variability in the experimental model,^14,15^ uncontrolled environmental influences,^16-18^ procedural errors,^19^ and occasionally, opportunistic or background subclinical infections in experimental models.^9,20,21^

**Figure 1.**
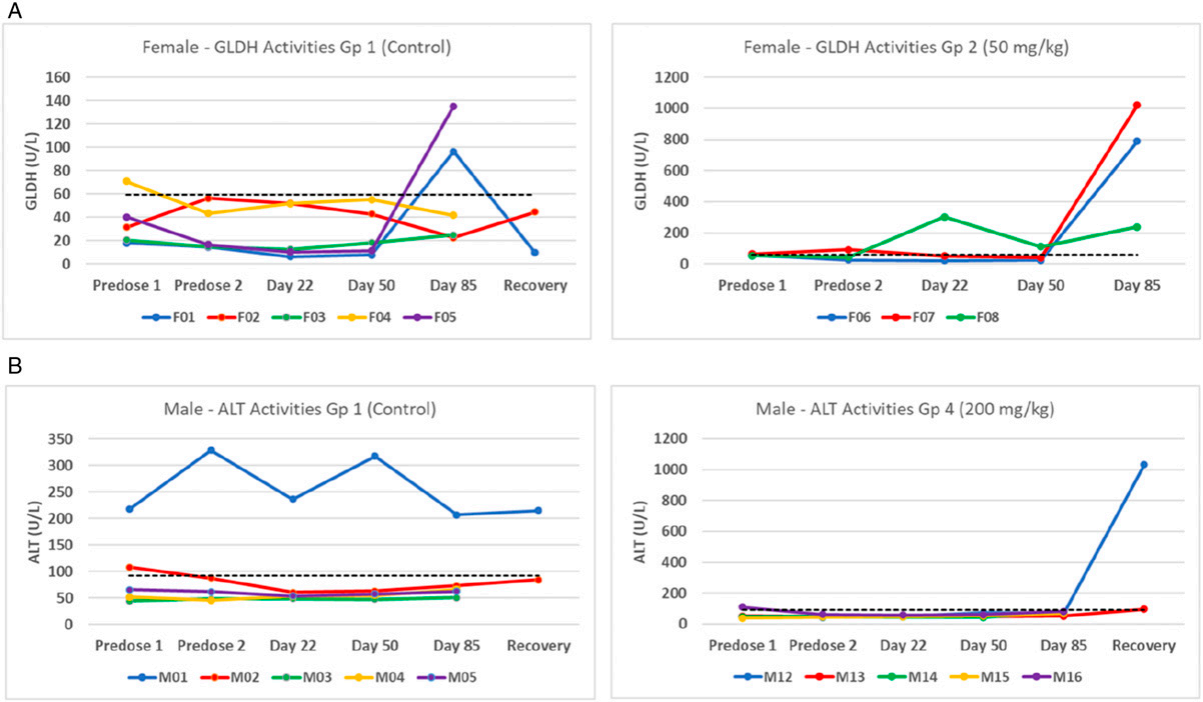
(A) Individual female animals showed variable increases in serum GLDH activities during 13 weeks of treatment. The dotted line represents the upper 97.5^th^ percentile value (59 U/L) in 460 control animals from 2012-2018. (B) Individual male serum ALT activities showed a single control animal (M01) with a persistently high enzyme activity level throughout the study and a single animal in Group 4 (200 mg/kg - M12) which had a markedly increased enzyme activity only following a 4-week treatment-free recovery period. The dotted line represents the upper 97.5^th^ percentile value (92.5 U/L) in 64 control animals from 2013-2014.

This report describes investigations that were prompted by unexpected observations in a toxicology study with a novel, orally-active, small molecule in clinical development for the treatment of a rare genetic disease. The molecule does not have immunomodulatory activity and there is no evidence that the pharmacodynamic activity would modulate susceptibility to infection (pers. com. sponsor company). During a 3-month toxicology study in 2020 with cynomolgus monkeys (*Macaca fascicularis* of Mauritian origin), at a well-established European contract laboratory, routine clinical pathology investigations showed that multiple animals, from all experimental groups including the controls, had serum enzyme activities aspartate transaminase (AST), glutamate dehydrogenase (GLDH), and alanine transaminase (ALT) which were above the normal range for the age and species of laboratory animal used (Figure 1A). In healthy individuals, the normal activity levels of these cytosolic enzymes is attributed to the level of cell renewal (turnover) in tissues which contain the highest level of these enzymes: liver, muscle, and intestine—and they are often collectively referred to as liver function tests (LFTs). When hepatocytes are injured, the activities of LFT enzymes usually increase in serum or plasma within 12-24 hours, with the magnitude of the increase giving a general indication of the extent of hepatocyte injury. In this study, the increased enzyme activities were not dose-related, nor obviously a direct consequence of treatment. Unusually, in some animals, serum enzyme activities were increased during a 4-week, off-treatment, recovery period (Figure 1B).

**Figure 2.**
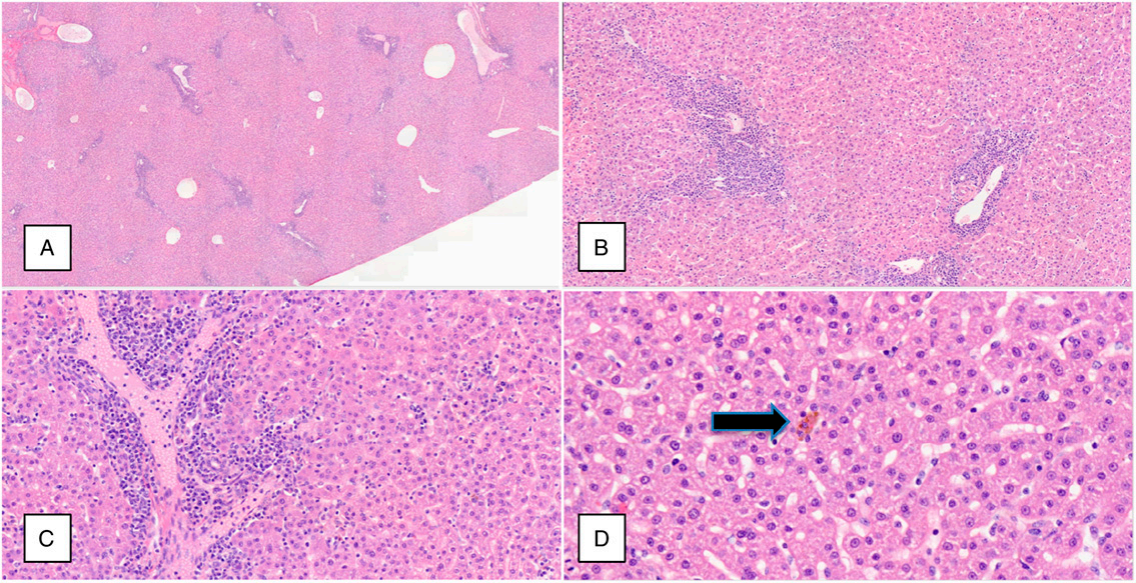
Hepatic histopathology (A) shows focal, predominantly periportal, mononuclear cell inflammatory infiltrates 1.5× magnification. Higher magnification (B = 5× magnification; C = 10× magnification) shows increased numbers of mononuclear cells in hepatic sinusoids (B and C) with pigment accumulation in an occasional hepatocyte or Kupffer Cell ((D) black arrow; 20× magnification). Animal M12 carriage return (200 mg/kg) following a 4-week off-treatment recovery period.

Histopathological examination of the liver revealed a minimal, slight, or moderate severity grade accumulation of mononuclear inflammatory cells, predominantly in the periportal zones of most animals, which is a frequent spontaneous observation in the liver of cynomolgus monkeys.^22,23^ Additionally, 10 of 42 animals from all treated and control groups had minimal or slight degeneration or necrosis of hepatocytes, frequently associated with slightly more marked chronic inflammatory cell infiltrates (Figure 2). The group distribution of these changes was unusual (i.e. absence of a dose relationship, high individual variability within a treatment group and control animals affected), prompting further questions.

**Figure 3.**
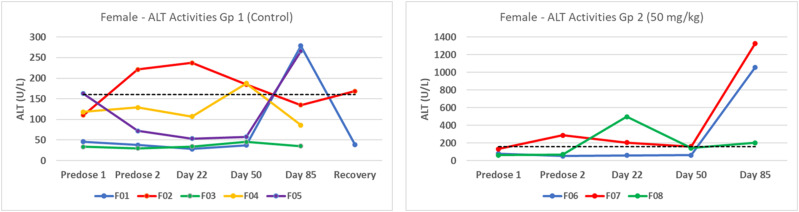
Serum ALT activities in female animals, showed unusually variable activity levels in control animals and, in two low dose (50 mg/kg) individuals, a significant increase at the end of the study. The dotted line represents the upper 97.5^th^ percentile value (160 U/L) in 532 control animals from 2012-2018.

Because the clinical pathology and hepatic pathology observations were more marked in some female animals treated with the lowest dose level than in the other groups, including controls (Figure 3), the draft toxicology report initially concluded that: “…the magnitude of the effects in ALT, GLDH, and AST activities in treated animals exceeded those of control animals, therefore, the enzyme level increase in treated animals were considered to be related to the test compound… Due to the adverse microscopic findings of increased mononuclear cell infiltrates in the liver of some of the animals administered ≥50 mg/kg, a no observed adverse effect level (NOAEL) was not determined for this study.”

Based on this interpretation, the drug was assessed as being hepatotoxic at all four dose levels tested, implying that a further study was needed to identify a NOAEL before clinical trials could continue. This initial assessment had potentially serious consequences for establishing a suitable starting dose level for the intended clinical trial. However, the unusual pattern of hepatotoxicity (some untreated control animals with enzyme activity levels above the upper limit of normal, distinct absence of any dose response, and markedly increased enzyme activity levels in individual animals after the withdrawal of treatment), prompted an alternative hypothesis: that a factor other than exposure to the test compound could be responsible for the effects in the liver. This paper documents the approach taken to address this question.

## Materials and Methods

The 3-month and 6-month toxicology studies described herein were conducted according to established pharmaceutical development guidelines^24^ by experienced contract laboratories in accordance with the OECD principles of Good Laboratory Practice^25^ and were fully compliant with National ethical and animal welfare guidelines.^26^

The cynomolgus monkeys used in the 3-month toxicology study were purpose-bred, all obtained from the same reputable source and were of Mauritian origin. Male animals were from a single delivery to the laboratory and female animals were from different deliveries. After leaving the breeding colony, they were transported, via an interim holding unit, to the contract laboratory. The study started in 2020 and, before allocation to the study, the health status of all animals was assessed by a veterinarian. Screening included a test for tuberculosis, although, as was normal practice at the laboratory, the animals were neither serologically screened nor vaccinated against HAV. Animals were housed in pairs or in treatment groups in a climate-controlled room, with a minimum of eight air changes/hour. Temperature range was 19 to 25°C and relative humidity 40 to 70%. Lighting was controlled to give a cycle of 12 hours of light and 12 hours of darkness. Certified lab diet was provided twice/day, supplemented with fresh fruit and vegetables. Water was provided ad libitum. Animals were acclimated to the study environment and procedures for at least 2 weeks before the study start. The test compound was formulated at weekly intervals in the vehicle 0.5% hydroxy-propyl-methyl-cellulose. Stability testing confirmed that the formulation of the test compound was stable for at least a week. Animals were administered the test compound orally, by gavage, twice weekly for 3 months. Control animals received the vehicle only, at the same dose volume (5 mL/kg). The study design is outlined in Table 1.

**Table 1.** Design of 3-Month Repeat Dose Toxicology Study in Unvaccinated Cynomolgus Monkeys.

Group Number	Group Description	Dose Level (mg/kg)	Dose Volume^a^ (mL/kg)	Animals/Group		Necropsy After	
				Males	Females	13 Weeks	17 Weeks
1	Control	0	5	5	5	3M/3F	2M/2F
2	Low	50	5	3	3	3M/3F	—
3	Intermediate low	100	5	3	3	3M/3F	—
4	Intermediate high	200	5	5	5	3M/3F	2M/2F
5	High	300	5	5	5	3M/3F	2M/2F

F = females; M = males.

^a^Based on most recent individual body weight.

Bodyweights of animals at the start of the study were 2.4-4.7 kg for males and 2.8-7.7 kg for females. Males were adolescent and females were sexually mature. Animals were group housed by treatment. Routine assessments included twice daily clinical examination, qualitative assessment of food consumption 1 day per week, weekly measurement of bodyweight, pre-treatment and pre-terminal of ophthalmoscopy, and recording of electrocardiogram and blood pressure. Serum samples for clinical pathology and blood samples for haematology analysis were freshly drawn on 2 occasions before dosing commenced, on Days 22, 50, and 85 during the treatment period, and after 4 weeks off treatment, which was the end of the recovery period. Assays were performed using validated analytical methods, with routine quality control samples to verify analytical performance. At termination of the animals at the end of the study, tissue samples were collected at post-mortem examination and processed using routine methods for histopathological analysis.^27^ In addition, sub-samples of liver (two pieces of approximately 2-4 g), were snap-frozen in a bath of solid carbon dioxide-cooled methanol and stored at −80°C.

Following the unusual changes in the profiles of some serum enzymes and slightly unusual liver histopathology, to investigate a possible infectious etiology of effects on the liver, samples of the snap-frozen liver (approximately 750 mg tissue) were retrieved from storage at −80C, transported frozen to an independent laboratory (Zoologix, Chatsworth, CA, USA) and tested for the presence of hepatitis viruses A, B, C, and E, using a proprietary highly specific Reverse Transcription-coupled, real time PCR method - Zoologix Test Codes S0242, S0243, S0244, S0245.^28,29^ Positive results were expressed as target copy number related to the level of expression of a house-keeping gene (β-actin RNA) in each of the same liver samples.

The experimental design and methods described for the 3-month toxicology study were broadly reproduced for the 6-month toxicology study (Table 2) with the notable difference that cynomolgus monkeys of Mauritian origin were vaccinated against HAV^30^ prior to their allocation to the study.

**Table 2.** Design of 6-Month Repeat Dose Toxicology Study in Vaccinated Cynomolgus Monkeys.

Group Number	Group Description	Dose Volume^a^ (mL/kg)	Animals/Group		Necropsy After	
			Males	Females	26 Weeks	34 Weeks
1	Control	5	6	6	4M/4F	2M/2F
2	Low	5	4	4	4M/4F	—
3	Intermediate low	5	4	4	4M/4F	—
4	Intermediate high	5	6	6	4M/4F	2M/2F
5	High	5	6	6	4M/4F	2M/2F

^a^Based on most recent bodyweight. M = male; F = female. Main study terminated on Day 185. Recovery period terminated on Day 239.

## Results

There was no evidence of infection with hepatitis viruses B, C, or E (data not shown). However, samples of liver from 20 of the 42 animals (48%) were strongly positive for hepatitis A virus (HAV) including 2 of 10 control group animals, with positive results ranging from 4 × 10^2^ to 7 × 10^4^ copies (Table 3). In individual animals, there was a strong association between confirmed HAV infection, increased serum activities of ALT, GLDH, AST and hepatocyte degeneration and/or necrosis. High activity levels of serum ALT activity were recorded at earlier timepoints in the study (Day 22 and 50) for 3 of 8 control animals which were not positive for HAV, suggesting that they may have recovered from an earlier infection. Therefore, 5 of 10 control animals were either positive for HAV at some time in the study or had unusually high serum ALT activity.

**Table 3.** Data From 3-Month Toxicology Study Show Strong Association Between Individual Animals With HAV Infection, Elevated Activity Levels of Serum ALT and GLDH, and Hepatic Pathology: Hepatocyte Degeneration/Necrosis And/or a Moderate Predominantly Periportal Inflammatory Infiltrate.

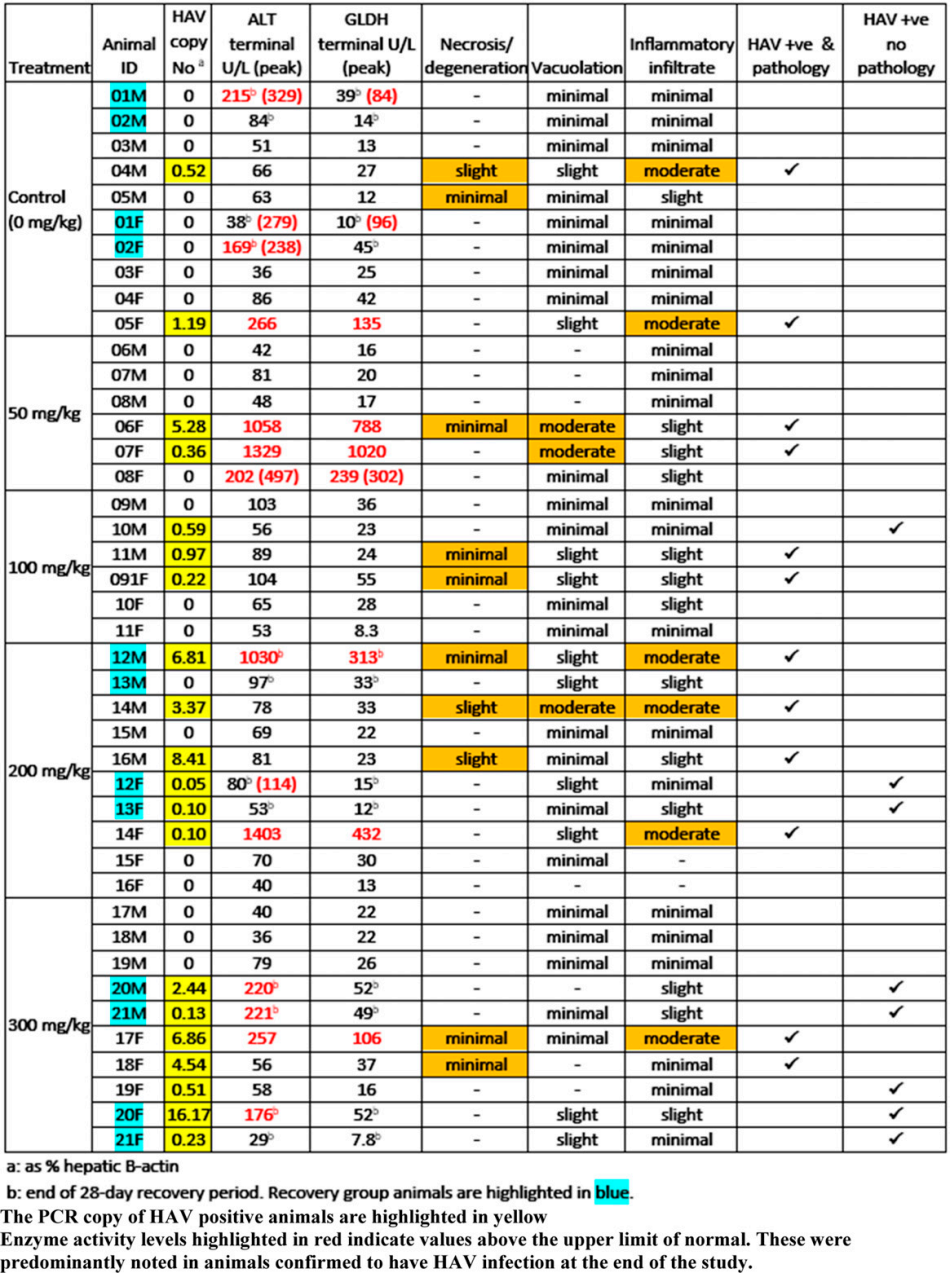

Overall, 13 of the 42 animals were reported to have hepatocyte necrosis, predominantly periportal micro-vesicular vacuolation, degeneration, and/or moderate mononuclear cell infiltrates—predominantly macrophage lineage, usually accompanied by high activities for serum ALT, AST, or GLDH (including some animals from each of the 5 different experimental groups), and 12 of these animals were confirmed to be positive for HAV infection (HAV copy number highlighted yellow, in Table 3). Generally, the animals with highest activity level of serum enzymes (indicating active hepatic injury), were positive for HAV. A few individuals which had high activity levels of serum enzymes ALT, AST, or GLDH at an earlier time in the study, on day 22 or day 50, did not test positive for the presence of HAV, at the end of the study, 10 weeks or more later, possibly indicating clearance of an earlier viral infection. Serological testing for antibodies against HAV antigens was not performed. Had this been conducted, one might have expected that animals which previously had an elevated activity level of serum transaminase activity, as a result of HAV infection, would show a significant titre of HAV-specific antibodies.

Re-evaluation of clinical pathology and histopathology data from this study in the light of the finding of HAV infection, led to the conclusion that the changes in the liver, which had initially been interpreted as drug induced hepatotoxicity, were in fact *not* caused by exposure to the test material, but were a consequence of subclinical viral infection. In retrospect, the hepatic pathology observed in this study was compatible with earlier reports of HAV infection in primates.^5,6^ In contrast to the initial interpretation, the final report revision then concluded that “*the compound was well tolerated at all dose levels and did not elicit any adverse effects.*” The highest dose level was the NOAEL. In hindsight, the observation before the start of the study, that several animals had pre-treatment serum enzyme activity levels above the upper limit of normal (above the 97.5^th^ percentile), should have prevented their inclusion in the study and prompted further investigation into exactly what was causing the elevated enzyme activity levels.

**Figure 4.**
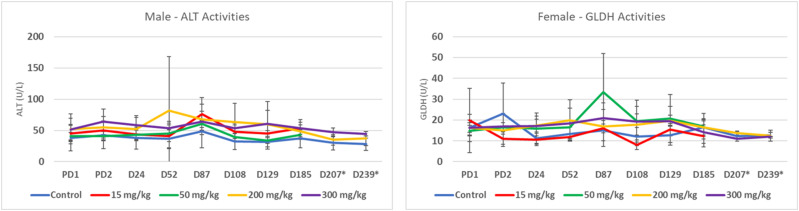
Serum ALT and GLDH profiles for males and females, respectively, for control and all 4-dose groups at 10 different time points during a 6-month toxicology study in cynomolgus monkeys. The 2-enzyme activity levels shown (ALT and GLDH) were chosen as the most sensitive indicators of hepatic impairment in the earlier 3-month toxicology study. Plots are mean +/− SD: PD = Predose. D = Day.

Despite initial uncertainty about the safety profile of this drug candidate in a non-human primate toxicology study of 3 months duration, it was eventually concluded that there were no adverse effects. This was subsequently confirmed in a 6-month toxicology study with the same drug candidate, given to HAV-vaccinated cynomolgus monkeys (see Table 2), at dose levels which bracketed the dose levels used in the earlier 3-month study. This study did not show any evidence of hepatotoxicity, as assessed by the most sensitive indicators of hepatic damage in the 3-month toxicology study (i) serum activity levels of ALT and GLDH (Figure 4) and (ii) by peer reviewed histopathology. Despite the potential for delay due to the elevated enzyme activities, the clinical development of this drug candidate has continued as scheduled.

## Discussion

Experience from this 3-month toxicology study in cynomolgus monkeys demonstrates that subclinical HAV infection can, and did, confound the preclinical safety evaluation of a drug candidate. In retrospect, based on this experience, if pre-experimental ALT activity levels in some individual animals are above the upper limit of normal (>95% confidence interval based on historic control data), then animals showing this pattern should be excluded from the study, or at least serologically screened to investigate evidence of an earlier exposure to an infectious agent. In the absence of routine serological or PCR screening for viral infection, one possible consequence is that a subclinical infection, could influence historical control data for clinical pathology or haematology parameters—as they may unwittingly include data from infected individuals. If this was the case, the upper limit of historic control data could be unrepresentatively high. Nonetheless, when historic control data is based on several hundred individuals, a few aberrant results should have little impact on its overall value as a reference point.

If, on completion of a study, an unusual pattern of enzyme increases is observed—particularly if an effect appears to worsen following an off-treatment recovery period—then careful follow up investigations for potentially confounding factors, including subclinical infection, should be considered. While rarely specifically investigated, subclinical HAV infection has occasionally been reported in laboratory housed primates,^8,9,31^ including those used for regulatory toxicology studies.^5^ Because the liver is the most frequent target organ for drug induced toxicity,^32,33^ it is particularly likely, that if viral hepatitis was to occur in a toxicology study, the clinical pathology and histopathology findings in animals could be erroneously interpreted (as initially in this study) as test compound-related effects. This possibility can be avoided if animals are vaccinated against HAV – as were the cynomolgus monkeys used in the subsequent 6-month toxicology study with this test compound.

Small foci of chronic inflammatory cells are frequently observed in periportal areas of the liver of rodents and non-human primates used in toxicology studies.^22,23^ When the incidence and severity grade of these lesions is low, with no obvious difference between the control and treated groups, they are normally dismissed as background lesions and may not even be reported if their size and occurrence is assessed to be within the normal spectrum for animals at that laboratory.

The highest levels of drug exposure in toxicology studies are commonly an order of magnitude or more greater than those intended for therapeutic use. Such high exposure levels are commonly recognised to cause a degree of physiological stress in the animals, sometimes evidenced by hypertrophy of steroid producing cells in the adrenal cortex, or by involution of the thymus or other lymphoid tissues.^34,35^ When high-dose stress effects are immunosuppressive, a pre-existing subclinical infection can become clinically evident,^36^ or in some instances, may be detectable via abnormal clinical pathology parameters. If a dose-related increase in stress results, this can create the false impression of a dose-related toxic effect, whereas it is, in fact, a treatment-related exacerbation of a subclinical infection, consequent to stress-related immunosuppression.

Zoonotic infection with hepatitis A or B viruses is a theoretical occupational health hazard for staff who work in experimental animal facilities^37,38^ and this can include staff who are responsible for maintaining the services of the vivarium (service and maintenance engineers), as well as the animal husbandry technicians, veterinarians, and staff who are in frequent and direct contact with the infected animals. To minimise this risk, in many laboratories, staff in regular contact with experimental animals are required to be vaccinated against potentially zoonotic infections.

Informal scientific communication and a recent report^39^ identified HAV infection in cynomolgus monkeys of Mauritian origin. In contrast to our experience, the recent report indicates that available PCR and serological tests (methods not stated) were not able to detect HAV infection in blood from infected animals, despite raised activity levels of ALT and GLDH. A PCR method was subsequently developed to detect this genotype of HAV infection, although it was reported that only 3.6% of (144 of 4,000) blood samples were positive—a different result from almost 50% of the liver samples (20 of 42) in our study. Based on these data, it is possible that the incidence of HAV infection in toxicology studies may have been higher than the scientific literature presently indicates and, that other toxicology studies may have been confounded.

The increased incidence of background infections in non-human primates, such as cynomolgus monkeys, may be related to the recent acute shortage of non-human primates for biomedical research, due to their non-availability from the normal major source of supply, China, since the outbreak of the COVID-19 pandemic.^40,41^ As a consequence of these restrictions, it is possible that animals which would not previously have been considered suitable for experimental use may have been allocated to studies. In the absence of systematic retrospective or prospective profiling of laboratory animals for hepatitis viruses, the true incidence of subclinical infection is unknown.

It is reasonable to infer that in some cases a background level of HAV infection could lead to an inappropriate decision to end the development of a potentially effective drug candidate. To avoid this possibility, it would be prudent to ensure that *all* primates used in regulatory toxicology studies have been effectively vaccinated against hepatitis viruses,^30^ and it is perhaps remarkable that this was not a universal practice in 2020.^39^

## Supplemental Material

Supplemental Material - Hepatitis A Virus Infection in Cynomolgus Monkeys Confounds the Safety Evaluation of a Drug Candidate Supplemental Material for Hepatitis A Virus Infection in Cynomolgus Monkeys Confounds the Safety Evaluation of a Drug Candidate by Chris J. Powell John C. Kapeghian, John C. Bernal, and John R. Foster in International Journal of Toxicology
