# Supplementary material for: Hepatitis A Virus Infection in Cynomolgus Monkeys Confounds the Safety Evaluation of a Drug Candidate
Source: Int J Toxicol. 2024 Mar 19;43(4):368–76. doi: 10.1177/10915818241237992 (PMC11155213; doi:10.1177/10915818241237992)
Supplement: Supplemental Material - Hepatitis A Virus Infection in Cynomolgus Monkeys Confounds the Safety Evaluation of a Drug Candidate [file sj-pdf-1-ijt-10.1177_10915818241237992.pdf]

Supplementary Data for:

Hepatitis A Virus Infection in Cynomolgus Monkeys Confounds the Safety Evaluation of a Drug Candidate

C J Powell et al

<u>13 week intermittent dosing cynomolgus monkey study – liver enzyme data and additional plots.</u>

Female GLDH Activity Levels for all dose groups

		U/L					
	Animal	Predose 1	Predose 2	Day 22	Day 50	Day 85	Recovery
Female - GLDH Activity Levels Gp 1 (Control)	F01	17.84	14.43	6.11	7.78	96.36	9.67
	F02	31.38	56.21	51.97	42.7	22.39	44.56
	F03	20.44	14.83	12.57	18.23	24.72	
	F04	70.84	43.29	51.8	55.15	41.63	
	F05	40.14	16.58	10.14	11.42	135.12	
Female - GLDH Activity Levels Gp 2 (50 mg/kg)	F06	57.3	25.22	21.84	22.44	787.84	
	F07	63.08	91.42	51.06	40.56	1019.52	
	F08	52.3	42.7	301.8	110.92	238.68	
Female - GLDH Activity Levels Gp 3 (100 mg/kg)	F09	21.76	13.39	17.3	12.53	54.78	
	F10	12.48	14.97	13.71	12.44	28.15	
	F11	11.9	9.04	5.66	7.69	8.34	
Female - GLDH Activity Levels Gp 4 (200 mg/kg)	F12	25.46	19.94	31.98	24.53	27.87	14.88
	F13	22.45	16.4	14.91	10.26	12.2	11.99
	F14	15.12	9.7	10.5	8.74	432.32	
	F15	12.43	8.94	29.56	18.53	30.18	
	F16	18.28	10.27	10.66	10.69	13.32	
Female - GLDH Activity Levels Gp 5 (300 mg/kg)	F17	22.83	35.25	23.87	38.53	105.7	
	F18	19.62	14.59	12.64	11.07	37.09	
	F19	24.71	13.99	12.39	10.48	16.24	
	F20	12.89	25.27	19.44	31	53.07	52.15
	F21	7.82	8.54	6.11	6.7	9.01	7.84

Sampling Occasion vs GLDH Level Plots for all Female Dose Groups (including data/plots shown in Figure 1A; The dotted line represents the upper 97.5th percentile value (59 U/L) in 460 control animals from 2012-2018)

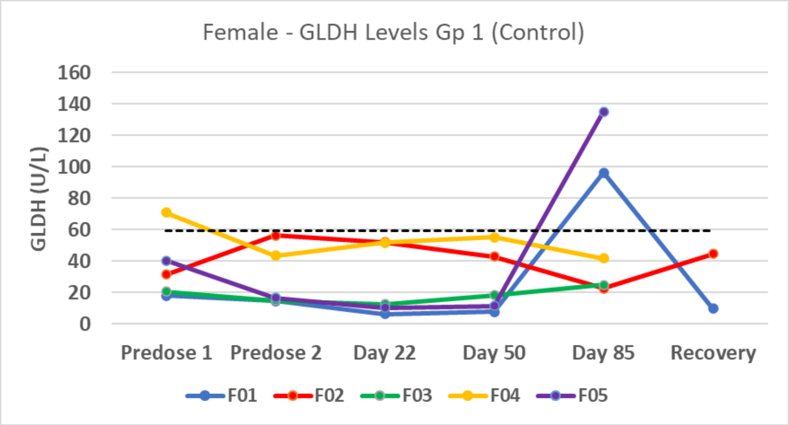

Female - GLDH Levels Gp 1 (Control)
GLDH (U/L)
160
140
120
100
80
60
40
20
0
Predose 1
Predose 2
Day 22
Day 50
Day 85
Recovery
F01
F02
F03
F04
F05

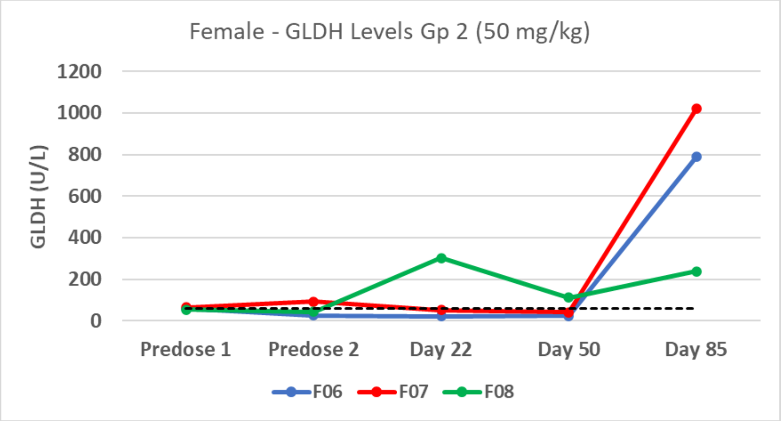

Female - GLDH Levels Gp 2 (50 mg/kg)
GLDH (U/L)
1200
1000
800
600
400
200
0
Predose 1
Predose 2
Day 22
Day 50
Day 85
F06
F07
F08

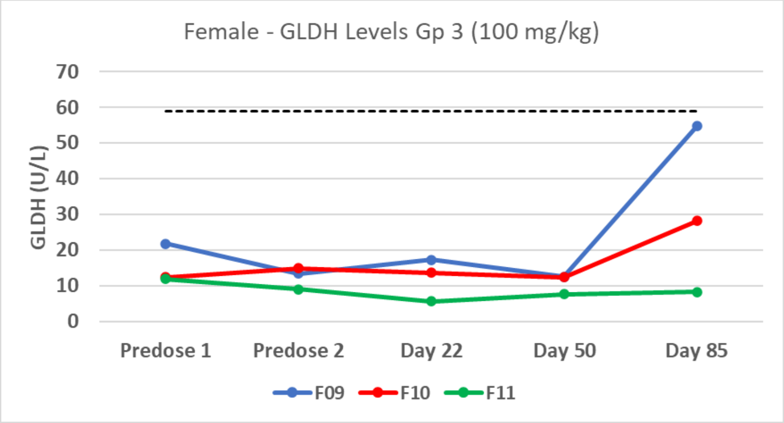

Female - GLDH Levels Gp 3 (100 mg/kg)
GLDH (U/L)
70
60
50
40
30
20
10
0
Predose 1
Predose 2
Day 22
Day 50
Day 85
F09
F10
F11

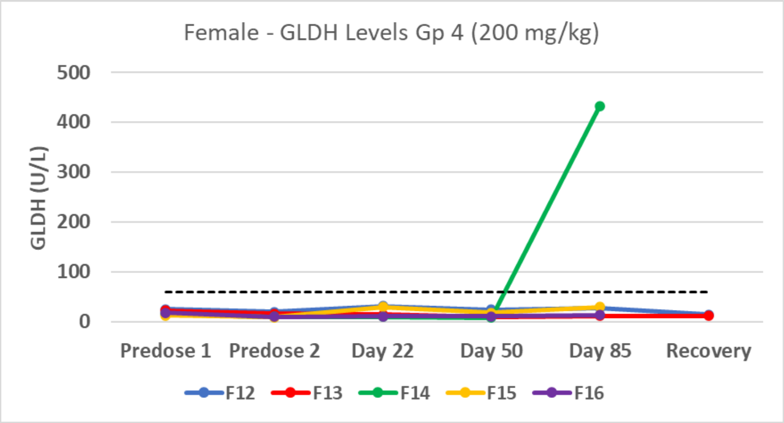

Female - GLDH Levels Gp 4 (200 mg/kg)
GLDH (U/L)
500
400
300
200
100
0
Predose 1
Predose 2
Day 22
Day 50
Day 85
Recovery
F12
F13
F14
F15
F16

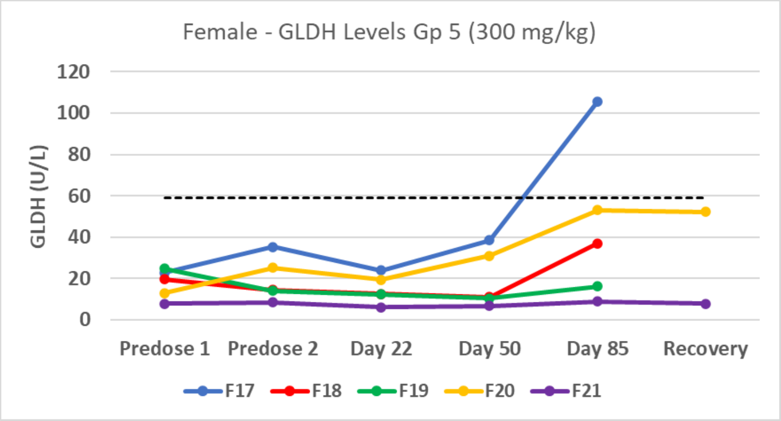

Female ALT Activity Levels for all dose groups

		U/L					
	Animal	Predose 1	Predose 2	Day 22	Day 50	Day 85	Recovery
Female - ALT Activity Levels Gp 1 (Control)	F01	45.96	37.88	28.49	37.5	278.69	38.36
	F02	110.16	221.43	237.54	185.06	134.89	169.01
	F03	33.66	29.9	33.99	45.18	35.51	
	F04	118.32	129.05	107	187.64	86.01	
	F05	162.84	72.3	53.28	57.66	265.94	
Female - ALT Activity Levels Gp 2 (50 mg/kg)	F06	78.73	53.52	59	63.08	1058.12	
	F07	131.95	288.17	203.86	160.55	1329	
	F08	62.91	68.76	496.92	143.13	201.88	
Female - ALT Activity Levels Gp 3 (100 mg/kg)	F09	43.54	43.37	55.81	38.59	103.87	
	F10	39.03	49.74	51.58	52.36	64.95	
	F11	47.43	41.22	37.03	44.07	52.7	
Female - ALT Activity Levels Gp 4 (200 mg/kg)	F12	109.95	74.99	113.16	89.85	95.37	80.24
	F13	40.75	46.47	54.35	35.2	51.65	52.74
	F14	30.36	28.99	34.69	34.28	1402.76	
	F15	42.67	44.42	67.12	50.38	69.72	
	F16	45.71	35.84	36.8	36.8	40.36	
Female - ALT Activity Levels Gp 5 (300 mg/kg)	F17	48.76	137.92	94.46	135.25	256.57	
	F18	33.29	30.08	29.69	27.29	55.6	
	F19	68.12	59.16	57	43.68	57.89	
	F20	25.47	31.3	31.16	46.5	80.44	175.8
	F21	14.89	17.7	21	21.99	53.17	28.93

Sampling Occasion vs ALT Level Plots for all Female Dose Groups (including data/plots shown in Figure 3; The dotted line represents the upper 97.5th percentile value (160 U/L) in 532 control animals from 2012-2018)

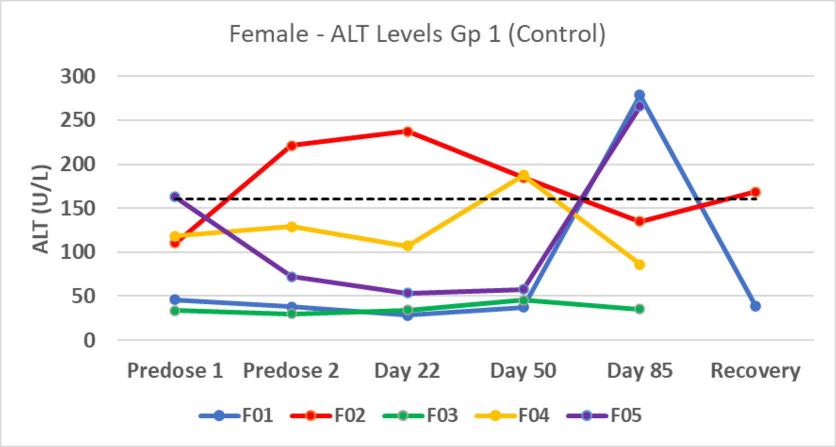
Female - ALT Levels Gp 1 (Control)
300
250
200
150
100
50
0
ALT (U/L)
Predose 1
Predose 2
Day 22
Day 50
Day 85
Recovery
F01
F02
F03
F04
F05

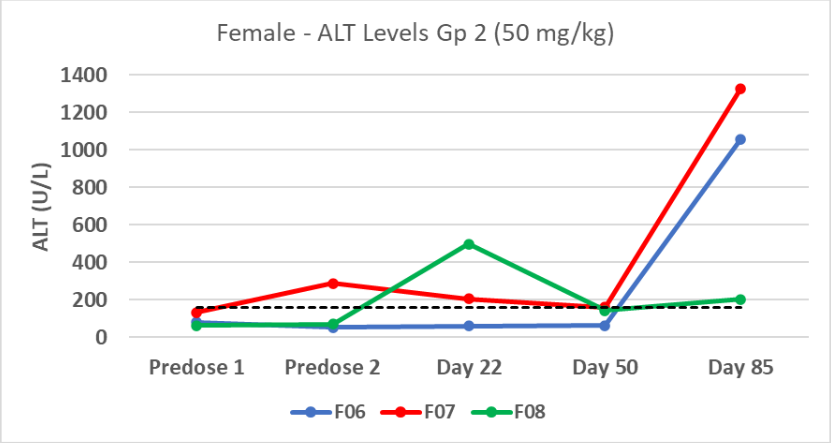
Female - ALT Levels Gp 2 (50 mg/kg)
1400
1200
1000
800
600
400
200
0
ALT (U/L)
Predose 1
Predose 2
Day 22
Day 50
Day 85
F06
F07
F08

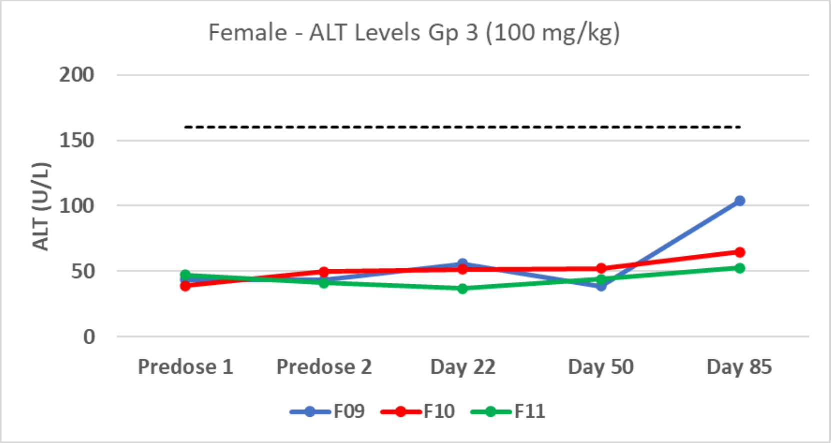
Female - ALT Levels Gp 3 (100 mg/kg)
200
150
100
50
0
ALT (U/L)
Predose 1
Predose 2
Day 22
Day 50
Day 85
F09
F10
F11

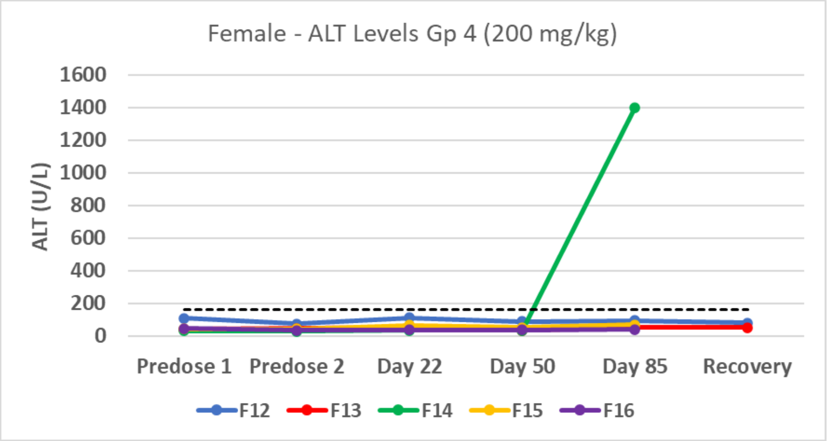
Female - ALT Levels Gp 4 (200 mg/kg)
1600
1400
1200
1000
800
600
400
200
0
ALT (U/L)
Predose 1
Predose 2
Day 22
Day 50
Day 85
Recovery
F12
F13
F14
F15
F16

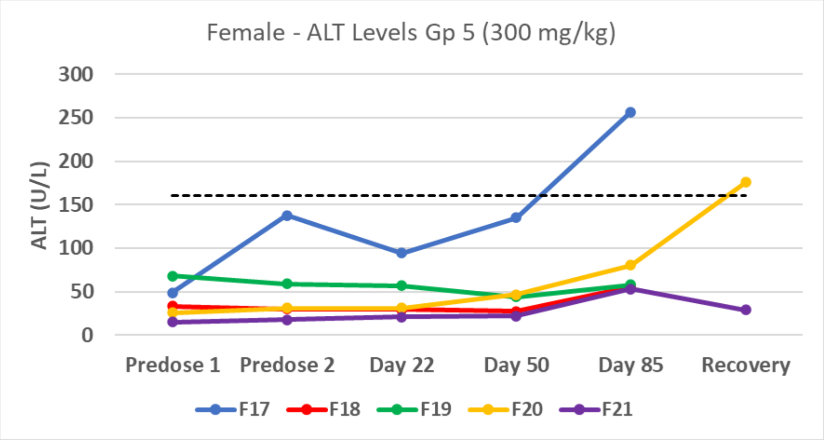

Male GLDH Activity Levels for all dose groups

		U/L					
	Animal	Predose 1	Predose 2	Day 22	Day 50	Day 85	Recovery
Male - GLDH Activity Levels Gp 1 (0 mg/kg)	M01	41.33	83.58	48.54	75.91	28.76	39.23
	M02	17.18	14.52	9.9	12.34	11.44	13.75
	M03	12.85	16.14	16.99	20.16	12.93	
	M04	20.59	17.94	22.01	29.92	27.38	
	M05	15.39	14.19	11.26	14.63	11.91	
Male - GLDH Activity Levels Gp 2 (50 mg/kg)	M06	19.53	19.2	18.53	19.15	16.24	
	M07	17.24	25.02	24.39	29.48	20.02	
	M08	16.59	16.17	12.17	14.57	17.44	
Male - GLDH Activity Levels Gp 3 (100 mg/kg)	M09	28.16	32.48	54.38	58.41	35.81	
	M10	26.41	26.8	24.7	25.48	22.8	
	M11	19.05	27.52	23.87	16.65	23.76	
Male - GLDH Activity Levels Gp 4 (200 mg/kg)	M12	13.91	13.48	12	40.3	22.13	313.44
	M13	18.29	21.12	17.13	21.75	15.53	32.8
	M14	11.9	9.71	9.05	13.21	33.3	
	M15	13.39	14.97	19.39	23.22	21.73	
	M16	13.58	12.8	14.21	21.46	22.95	
Male - GLDH Activity Levels Gp 5 (300 mg/kg)	M17	13.65	21.96	15.19	25.77	21.9	
	M18	21.52	26.57	30.58	57.9	21.9	
	M19	22.01	24.1	20.38	17.27	26.02	
	M20	19.09	17.55	23.82	23.63	19.95	52.98
	M21	19.41	14.26	17.43	20.71	15.82	48.93

Sampling Occasion vs GLDH Level Plots for all Male Dose Groups. The dotted line represents the upper 97.5th percentile value (59 U/L) in 460 control animals from 2012-2018.

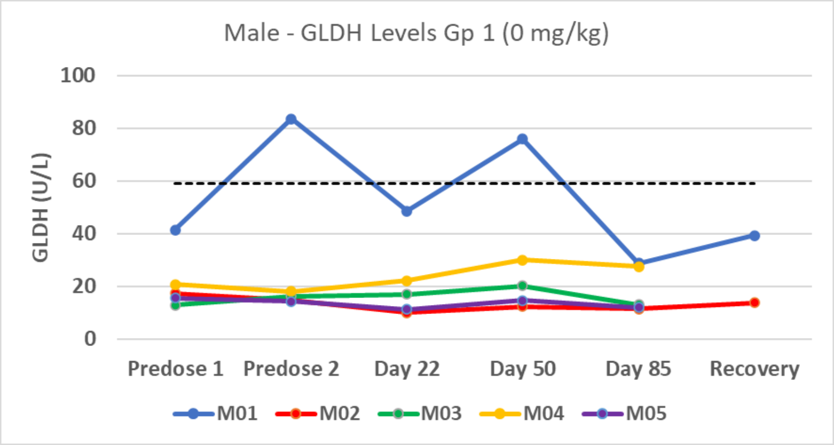

Male - GLDH Levels Gp 1 (0 mg/kg)
GLDH (U/L)
100
80
60
40
20
0
Predose 1
Predose 2
Day 22
Day 50
Day 85
Recovery
M01
M02
M03
M04
M05

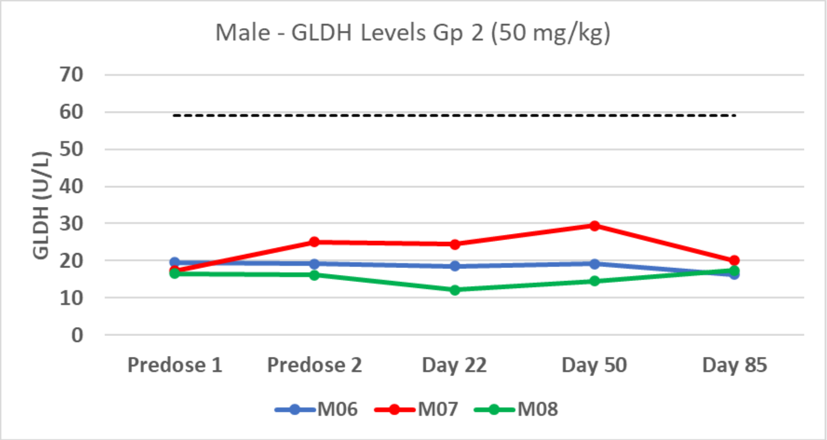

Male - GLDH Levels Gp 2 (50 mg/kg)
GLDH (U/L)
70
60
50
40
30
20
10
0
Predose 1
Predose 2
Day 22
Day 50
Day 85
M06
M07
M08

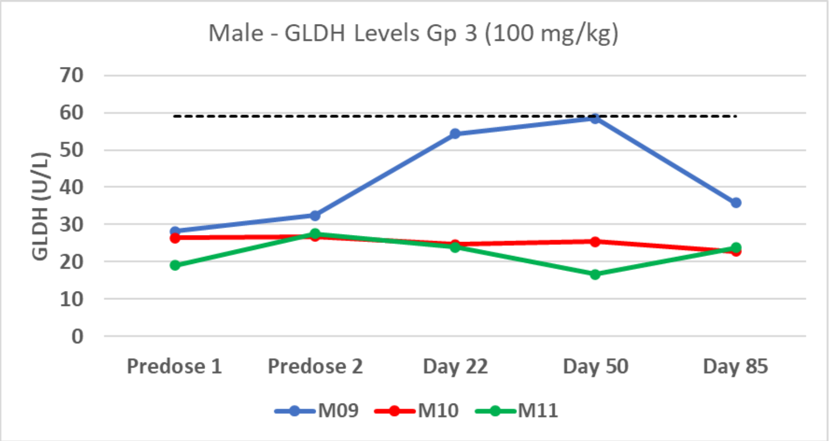

Male - GLDH Levels Gp 3 (100 mg/kg)
GLDH (U/L)
70
60
50
40
30
20
10
0
Predose 1
Predose 2
Day 22
Day 50
Day 85
M09
M10
M11

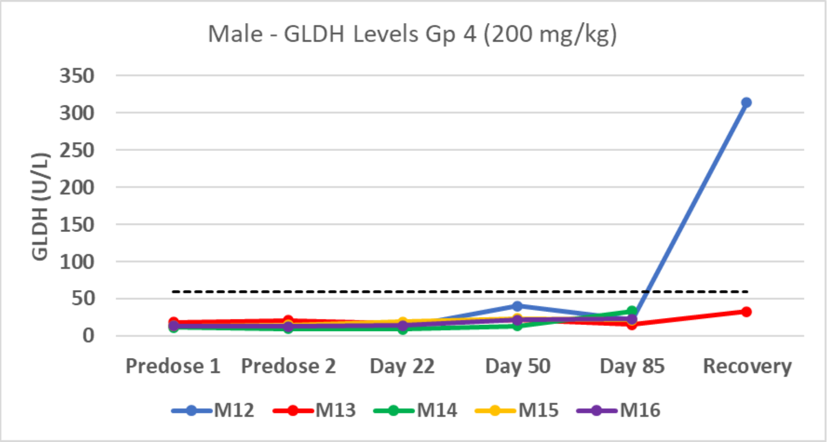

Male - GLDH Levels Gp 4 (200 mg/kg)
GLDH (U/L)
350
300
250
200
150
100
50
0
Predose 1
Predose 2
Day 22
Day 50
Day 85
Recovery
M12
M13
M14
M15
M16

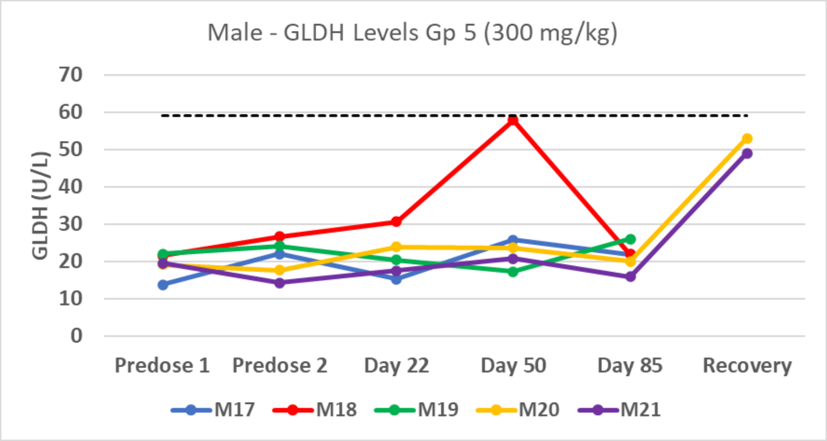

Male ALT Activity Levels for all dose groups

		U/L					
	Animal	Predose 1	Predose 2	Day 22	Day 50	Day 85	Recovery
Male - ALT Activity Levels Gp 1 (Control)	M01	217.44	328.96	236.2	317.77	206.7	214.71
	M02	108.16	87.37	60.4	62.77	73.05	83.98
	M03	44.08	47.8	48.49	47.28	50.5	
	M04	51.95	45.43	52.84	55.05	66.22	
	M05	65.25	61.52	53.78	57.5	62.7	
Male - ALT Activity Levels Gp 2 (50 mg/kg)	M06	49.41	48.87	50.41	42.3	42.44	
	M07	75.78	77.76	81.28	76.91	81.26	
	M08	42.28	46.09	35.19	34.81	48.11	
Male - ALT Activity Levels Gp 3 (100 mg/kg)	M09	78.02	72.31	91.28	88.4	102.99	
	M10	67.18	58.88	52.35	52.18	56.44	
	M11	82.06	105.79	94.14	44.52	88.99	
Male - ALT Activity Levels Gp 4 (200 mg/kg)	M12	48.05	43.78	47.34	73.48	69.98	1030.8
	M13	51.92	49.86	51.45	49.22	51.53	96.87
	M14	47.46	47.1	44.29	42.05	77.75	
	M15	37.91	44.46	47.72	50.81	69.12	
	M16	110.3	61.5	56.96	59.56	81.27	
Male - ALT Activity Levels Gp 5 (300 mg/kg)	M17	35.59	39.9	33.92	43.49	40.28	
	M18	39.69	38.08	36.7	59.93	35.92	
	M19	72.3	71.19	59.05	51.7	78.73	
	M20	84.61	68.44	75.4	66.2	75.34	220.43
	M21	52.83	48.52	48.82	44.47	36.87	220.61

Sampling Occasion vs ALT Level Plots for all Male Dose Groups (including data/plots shown in Figure 1B; The dotted line represents the upper 97.5th percentile value (92.5 U/L) in 64 control animals from 2013-2014)

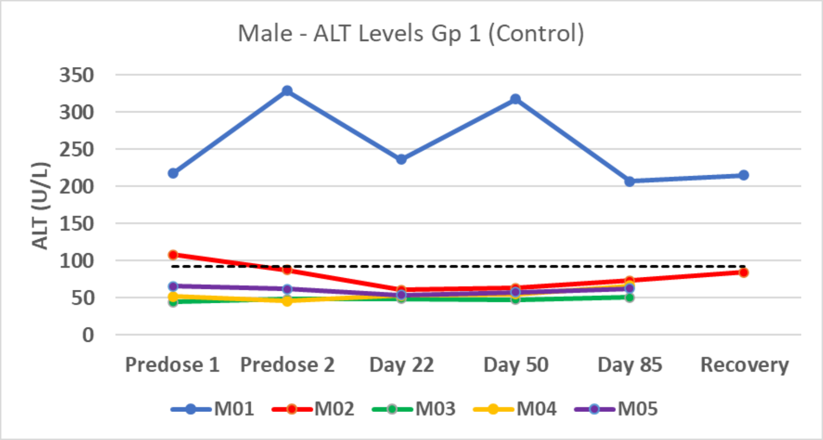

Male - ALT Levels Gp 1 (Control)
ALT (U/L)
350
300
250
200
150
100
50
0
Predose 1
Predose 2
Day 22
Day 50
Day 85
Recovery
M01
M02
M03
M04
M05

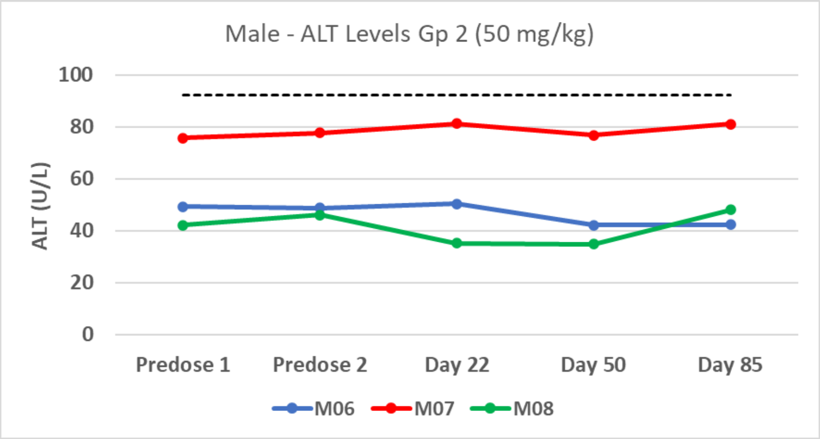

Male - ALT Levels Gp 2 (50 mg/kg)
ALT (U/L)
100
80
60
40
20
0
Predose 1
Predose 2
Day 22
Day 50
Day 85
M06
M07
M08

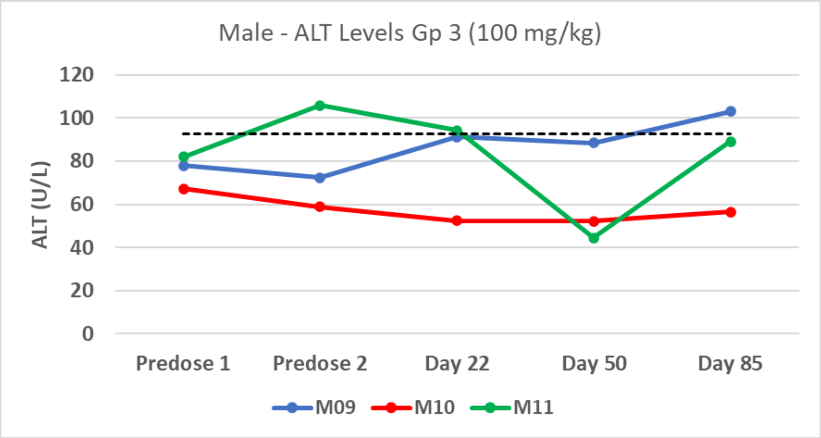

Male - ALT Levels Gp 3 (100 mg/kg)
ALT (U/L)
120
100
80
60
40
20
0
Predose 1
Predose 2
Day 22
Day 50
Day 85
M09
M10
M11

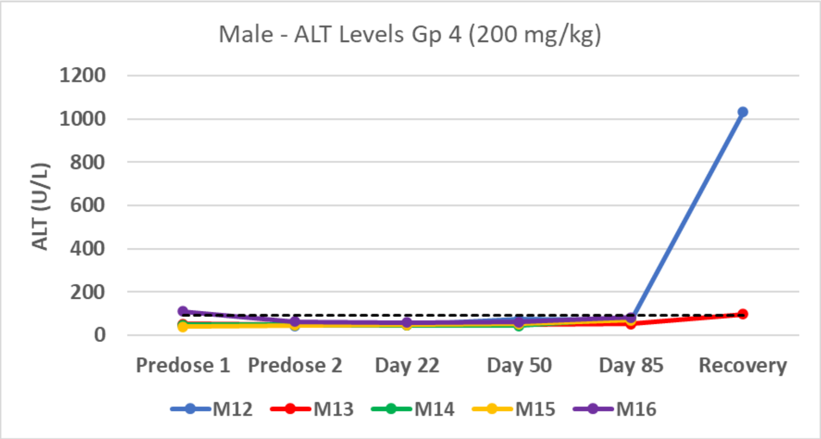

Male - ALT Levels Gp 4 (200 mg/kg)
ALT (U/L)
1200
1000
800
600
400
200
0
Predose 1
Predose 2
Day 22
Day 50
Day 85
Recovery
M12
M13
M14
M15
M16

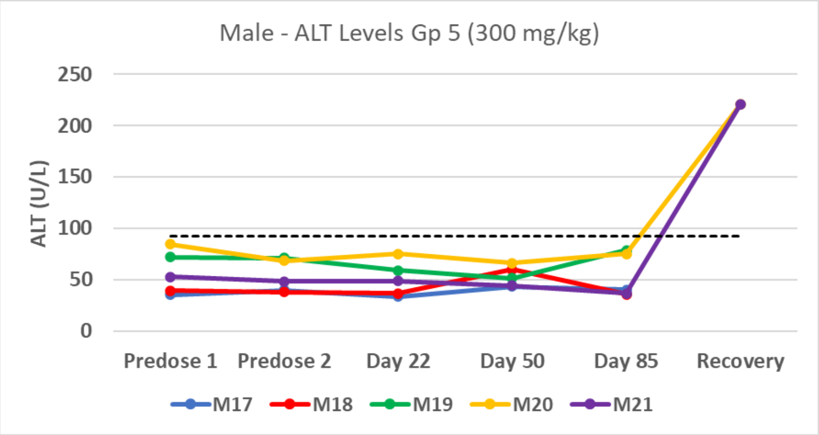

__**26 week intermittent dosing cynomolgus monkey study – liver enzyme data**__

Male Mean +/- Standard Deviation (SD) ALT Activity Levels for all dose groups/sampling occasions (PD = Predose; D = Day); Plot Shown in Figure 5.

		U/L									
		PD1	PD2	D24	D52	D87	D108	D129	D185	D207	D239
Male - ALT Activity Levels Gp 1 (Control)	Mean	38.2	42.3	37.8	36.5	48.7	32.3	32.0	37.3	30.0	28.0
	SD	21.0	21.6	17.7	14.6	26.7	13.1	11.7	15.1	11.3	9.9
	N	6	6	6	6	6	6	6	6	2	2
Male - ALT Activity Levels Gp 2 (15 mg/kg)	Mean	45.0	50.0	43.5	41.0	76.3	48.0	45.3	53.7		
	SD	13.2	15.2	9.6	20.5	26.2	10.8	4.5	9.5		
	N	4	4	4	4	3	3	3	3		
Male - ALT Activity Levels Gp 3 (50 mg/kg)	Mean	41.0	40.8	43.0	45.3	60.3	39.3	33.8	43.3		
	SD	6.1	7.5	7.4	13.2	5.9	6.2	4.5	7.8		
	N	4	4	4	4	4	4	4	4		
Male - ALT Activity Levels Gp 4 (200 mg/kg)	Mean	52.2	55.0	52.3	81.5	67.8	63.2	59.7	48.8	35.0	37.0
	SD	23.9	17.2	18.8	86.7	24.2	30.5	36.4	10.2	8.5	11.3
	N	6	6	6	6	6	6	6	6	2	2
Male - ALT Activity Levels Gp 5 (300 mg/kg)	Mean	51.2	63.8	58.7	53.8	64.3	53.2	60.3	53.7	47.0	44.5
	SD	18.0	20.5	14.7	10.8	16.2	8.6	21.7	13.3	7.1	3.5
	N	6	6	6	6	6	6	6	6	2	2

Female Mean +/- Standard Deviation (SD) GLDH Activity Levels for all dose groups/sampling occasions (PD = Predose; D = Day); Plot Shown in Figure 5.

		U/L									
		PD1	PD2	D24	D52	D87	D108	D129	D185	D207	D239
Female - GLDH Activity Levels Gp 1 (Control)	Mean	16.2	23.0	11.2	13.3	14.9	12.1	12.7	16.5	12.3	12.7
	SD	6.5	14.7	2.4	3.2	5.0	4.4	4.7	3.7	2.4	1.4
	N	6	6	6	6	6	6	6	6	2	2
Female - GLDH Activity Levels Gp 2 (15 mg/kg)	Mean	19.8	11.0	10.6	11.9	16.2	8.1	15.5	12.3		
	SD	15.4	3.6	2.9	1.5	8.9	2.3	2.8	5.3		
	N	4	4	4	4	4	4	3	3		
Female - GLDH Activity Levels Gp 3 (50 mg/kg)	Mean	14.9	16.4	15.9	16.5	33.4	19.4	20.7	17.0		
	SD	2.5	2.2	7.6	3.3	18.6	10.1	11.5	6.1		
	N	4	4	4	4	4	4	4	4		
Female - GLDH Activity Levels Gp 4 (200 mg/kg)	Mean	16.3	15.0	17.3	19.8	16.9	17.8	19.6	16.4	13.7	12.5
	SD	2.3	1.9	3.1	9.9	1.2	5.1	2.6	4.2	0.9	2.6
	N	6	6	6	6	6	5	5	5	2	2
Female - GLDH Activity Levels Gp 5 (300 mg/kg)	Mean	16.4	17.0	17.1	18.4	21.0	19.3	19.5	14.2	11.0	12.1
	SD	3.6	6.1	4.8	7.3	7.2	7.2	7.2	5.4	1.3	0.1
	N	6	6	6	6	6	6	6	6	2	2
